# Validating a segment on chromosome 7 of *japonica* for establishing low-cadmium accumulating *indica* rice variety

**DOI:** 10.1038/s41598-021-85324-0

**Published:** 2021-03-15

**Authors:** Kai Wang, Tian-ze Yan, Shi-long Xu, Xu Yan, Qun-feng Zhou, Xin-hui Zhao, Yan-feng Li, Zhong-xiu Wu, Peng Qin, Chen-jian Fu, Jun Fu, Yan-biao Zhou, Yuan-zhu Yang

**Affiliations:** 1 Key Laboratory of Southern Rice Innovation and Improvement, Ministry of Agriculture and Rural Affairs, Hunan Engineering Laboratory of Disease and Pest Resistant Rice Breeding, Yuan Longping High-Tech Agriculture Co., Ltd., Changsha, 410128 China; 2 State Key Laboratory of Hybrid Rice, Hunan Hybrid Rice Research Center, Changsha, 410125 China; 3 Department of Botany, College of Life Sciences, Hunan Normal University, Changsha, 410081 China; 4 College of Plant Science and Technology, Huazhong Agricultural University, Wuhan, 430070 China

**Keywords:** Genetics, Plant sciences

## Abstract

Cadmium (Cd) contamination of rice is a serious food safety issue that has recently been gaining significant public attention. Therefore, reduction of Cd accumulation in rice grains is an important objective of rice breeding. The use of favourable alleles of Cd accumulating genes using marker-assisted selection (MAS) is theoretically feasible. In this study, we validated a segment covering *OsHMA3*-*OsNramp5*-*OsNramp1* on chromosome 7 of *japonica* for establishing low-cadmium accumulating *indica* rice variety. The *OsHMA3*-*OsNramp5*-*OsNramp1*^*jap*^ haplotype significantly decreased grain Cd concentration in middle-season *indica* genetic background. The improved 9311 carrying the *OsHMA3*-*OsNramp5*-*OsNramp1*^*jap*^ haplotype with recurrent parent genome recovery of up to 91.6% resulted in approximately 31.8% decrease in Cd accumulation in the grain and with no penalty on yield. There is a genetic linkage-drag between *OsHMA3*-*OsNramp5*-*OsNramp1 *^*jap*^ and the gene conditioning heading to days (HTD) in the early-season *indica* genetic background. Because the *OsHMA3*-*OsNramp5*-*OsNramp1-Ghd7*^*jap*^ haplotype significantly increases grain Cd concentration and prolongs growth duration, the linkage-drag between *OsHMA3*-*OsNramp5*-*OsNramp1* and *Ghd7* should be broken down by large segregating populations or gene editing. A novel allele of *OsHMA3* was identified from a wide-compatibility *japonica* cultivar, the expression differences of *OsNramp1* and *OsNramp5* in roots might contribute the Cd accumulating variation between *japonica* and *indica* variety.

## Introduction

Contamination of agricultural soils with heavy metals is a serious threat to crop production. Cadmium (Cd), one of the most dangerous heavy metals, has long been recognised as a major threat to human health through contamination of the food chain^1^. Rice (*Oryza sativa* L.) is the staple food for more than half of the world’s population, as well as a crop with high Cd accumulating abiltiy^2^. To suppress Cd pollution of food, rice has a strict upper level of Cd content. According to the Codex Alimentarius Commission of the FAO/WHO, the maximum Cd concentration in polished rice should not exceed 0.4 mg/kg, whereas stricter limits were implemented in China, with a maximum level for Cd of 0.2 mg/kg^3,4^. Rice-derived products are subject to the risk of Cd contamination, and consequent threats to consumer health if rice is grown in Cd-contaminated agricultural soil^5^. In some areas of China, Japan, Thailand, Bangladesh, Indonesia and Korea, the production of rice grains with high Cd levels, and subsequent transfer of the contaminant to the human food chain, is a serious environmental and food safety issue^6,7^. Fortunately, rice germplasm carries a large number of genetic variations related to grain Cd concentration. *Japonica* cultivars generally accumulate lower concentrations of Cd than *indica* and *aus* cultivars^2,8–10^, suggesting that Cd accumulation is genetically controlled in rice. It should, therefore, be possible to breed rice cultivars with a low Cd accumulation based on genetic improvement. Cd, from soil to grains in rice, has been a subject of considerable research and several relevant genes have been identified in recent years, including *OsIRT1*, *OsHMA3*, *OsNramp1*, *OsNramp5*, *OsHMA2*, *OsLCD* and *OsLCT1*^11–19^. Although genotypic differences in Cd concentration and several genes have been studied, only limited efforts have been made to breed rice with reduced Cd content, especially in *indica* cultivars.

Of the Cd transport-related genes of rice, *OsHMA3*, which is a major quantitative trait locus (QTL) mapped and cloned from low Cd-accumulating *japonica* cultivars (Akita 63 and Nipponbare), plays a transporter in Cd compartmentation into root cell vacuoles. Non-functional allele of *OsHMA3* results in high efficiency of root-to-shoot Cd translocation^12,13^. *OsNramp1* and *OsNramp5* are metal transporter genes that have been suggested to mediate the adsorption of Cd from soil to root^14–16^. The expression of *OsNRAMP1* in roots was found to be higher in high Cd-accumulating *indica* cultivars (Habataki, Anjana Dhan, Jarjan) than in low Cd-accumulating *japonica* varieties (Sasanishiki, Nipponbare)^14^. The expression of *OsHMA3* and *OsNRAMP5* in roots was lower in high Cd-accumulating *indica* cultivars (9311) than in low Cd-accumulating varieties, including PA64S, which was derived from an *indica*/*japonica* cross^20–22^. Variations in the promoter sequences was found between *indica* and *japonica* cultivars, which might lead to differences between in the expression levels of *OsHMA3*, *OsNRAMP1* and *OsNRAMP5*, as well as in Cd accumulation in the grains^14,20–22^. In addition, mutant *osnramp5* produced by carbonion-beam irradiation greatly decreases Cd uptake by roots, resulting in decreased Cd in grain and exhibit no agriculturally adverse traits^15^. The combined action of *OsHMA3*, *OsNRAMP1* and *OsNRAMP5* might contribute to the difference in grain Cd accumulation between *indica* and *japonica* varieties. Coincidentally, these three genes—*OsHMA3*, *OsNramp5* and *OsNramp1*—are all located on 7.4 Mb to 9.0 Mb of chromosome 7 within a region of about 1565 kb, according to the Rice Genome Annotation Project Database^23^. The segment covering *OsHMA3*-*OsNramp5*-*OsNramp1* from a low Cd-accumulating *japonica* cultivar may be valuable for improving *indica* varieties to produce low Cd accumulation.

The severity of rice grain Cd pollution in some areas of China led to the initiation of a national key breeding programme to reduce the Cd content of *indica* rice in 2014. This study was conducted with the objectives of (1) validating the effect of the *OsHMA3*-*OsNramp5*-*OsNramp1* region on chromosome 7 for grain Cd accumulation; (2) evaluating the use of *OsHMA3*-*OsNramp5*-*OsNramp1*^*jap*^ for *indica* improvement with low Cd accumulation in grains and (3) improving the elite *indica* cultivar with low Cd grain accumulation by MAS.

## Results

### Phenotypic variation and the effects of *OsHMA3*-*OsNramp5*-*OsNramp1* in recombinant inbred lines derived from an *indica*–*japonica* cross

**Figure 1:**
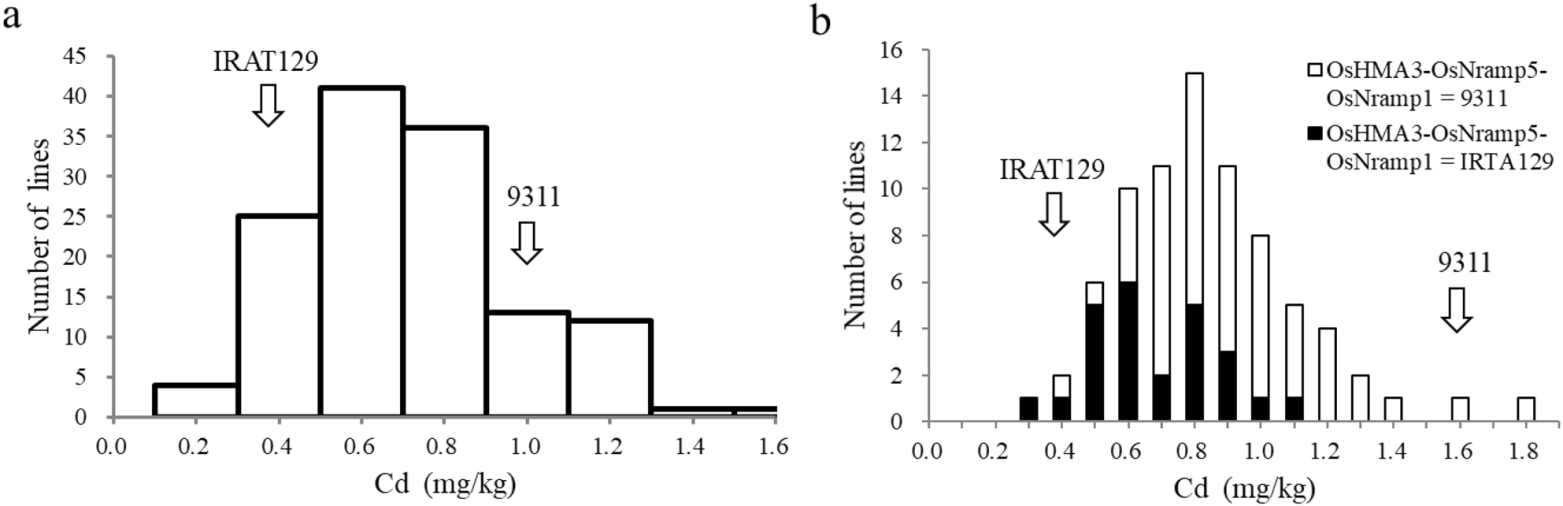
Phenotypic distributions of brown rice Cd concentration in RILs. (**a**) Distributions of brown rice Cd concentration of RILs in the 2016 middle-season rice growing season. (**b**) Distribution of brown rice Cd concentration and its association with the two genotypic groups of 78 lines from RILs across 2016 and 2017 middle-season rice growing season. *OsHMA3*-*OsNramp5*-*OsNramp* = 9311, carrying *OsHMA3*-*OsNramp5*-*OsNramp* from 9311; *OsHMA3*-*OsNramp5*-*OsNramp* = IRAT129, carrying *OsHMA3*-*OsNramp5*-*OsNramp* from IRAT129.

A recombinant inbred line (RIL) population was bred from crosses between *indica* variety 9311 and a *japonica* landrace IRAT129. The 9311 variety is an elite two-line middle-season *indica* hybrid restorer in China that accumulates Cd at high levels, and the IRAT129 variety is a high-compatibility *japonica* landrace that has a low ability to accumulate Cd. IRAT129, 9311, and RILs with 133 lines were grown in a Cd-polluted paddy field at Ningxiang in the 2016 middle-season rice growing season (Table 1). The Cd concentrations of IRAT129 and 9311 brown rice were 0.493 and 1.020 mg/kg, respectively. The segregation of Cd concentration in brown rice RILs was continuously distributed with Cd concentrations ranging from 0.133 to 1.463 mg/kg, averaging to 0.609 mg/kg (Fig. 1a). The kurtosis (0.174) and skewness (0.649) of the distribution of Cd concentrations were < 1, indicating a normal distribution. Transgressive segregation in both directions was observed, indicating predominance of additive gene action contributed by both parental alleles.

**Table 1:** Populations used to validate the effect of OsHMA3-OsNramp5-OsNramp1 from IRAT129. The IRAT129, 9311, H611 and H819 presented in the column of samples indicate the haplotype of *OsHMA3-OsNramp5-OsNramp1*.

Population	Generation	Segregating target genes	Samples	Growing season	Trait measured
RILs	F_6_		133 lines, containing 25 lines of IRAT129, 53 lines of 9311	Middle-season rice cropping season in 2016	Cd
	F_7_	*OsHMA3-OsNramp5-OsNramp1*	25 lines of IRAT129, 53 lines of 9311	Middle-season rice cropping season in 2017	Cd
NIL611	BC_3_F_2:3_		30 lines of IRAT129, 30 lines of H611 and 60 lines of heterozygote	Early-season rice cropping season in 2017	HTD, Cd
NIL819	BC_3_F_2:3_		30 lines of IRAT129, 28 lines of H819 and 60 lines of heterozygote		
NIL9311	BC_3_F_2:3_		23 lines of IRAT129, 30 lines of 9311 and 59 lines of heterozygote	Middle-season rice cropping season in 2017

A total of 133 RILs were genotyped using three pairs of intragenic molecular markers for *OsHMA3*, *OsNramp5* and *OsNramp1* genes (Table 2). Overall, 78 F_7_ lines with a homozygous genomic region covering *OsHMA3*, *OsNramp5* and *OsNramp1* genes (25 *OsHMA3*-*OsNramp5*-*OsNramp1*^*jap*^ homozygotes and 53 *OsHMA3*-*OsNramp5*-*OsNramp1*^*ind*^ homozygotes) were selected and their phenotypes were investigated in 2017. The frequency distribution of Cd concentration was not distinguishable among different genotypes, but *OsHMA3*-*OsNramp5*-*OsNramp1*^*jap*^ homozygous lines tended to have lower values and *OsHMA3*-*OsNramp5*-*OsNramp1*^*ind*^ homozygous lines had higher values (Fig. 1b). Results of a two-way ANOVA on the phenotypic differences between *OsHMA3*-*OsNramp5*-*OsNramp1*^*jap*^ and *OsHMA3*-*OsNramp5*-*OsNramp1*^*ind*^ homozygous lines in 2016 and 2017 are presented in Table 3. Significant variation was apparent between the two homozygous genotypic groups over the 2 years. Genotypes of *OsHMA3*-*OsNramp5*-*OsNramp1* explained 10.9% of the phenotypic variance in Cd concentration in 2016 and 8.6% of the variance in 2017. The *OsHMA3*-*OsNramp5*-*OsNramp1* haplotype of IRAT129 appeared to be associated with decreased Cd concentration in brown rice.

**Table 2:** Three developed intra-genic markers used to amplify Cd accumulating genes.

Gene	Marker	Primer sequence		Expected size (bp) of IRAT129 and 9311 (IRAT129/9311)	Restriction enzyme
		Forward (5′-3′)	Reverse (5′-3′)		
*OsHMA3*	HMA3	AATCCATCCAACCAAACCCGGAA	ATGCCCAGCGATCCAAGCTC	280 bp	BsaHI
*OsNramp5*	Nramp5	TTGTCATGCCGTACGCTT	CACCAAGGCAGAATGCAA	140/144 bp	
*OsNramp1*	Nramp1	GGCTGTAGATACCACATCACC	GCTGCCTCAAGAATTATACCTG	761/355 bp

**Table 3:** Brown rice Cd concentration and HTD of different genotypes differing in the *OsHMA3-OsNramp5-OsNramp1* region.

Population	Year	Trait	Phenotype of different genotypes (Mean ± SD)			ANOVA		A	D	D/[A]	*R*^2^
			*japonica*	*indica*	H	*F*	*P*				
RIL	2016	Cd	0.486 ± 0.186	0.694 ± 0.274	n.a	12.05	< .*0001*	0.10			10.9
	2017	Cd	0.769 ± 0.260	1.033 ± 0.372	n.a	10.07	< .*0001*	0.13			8.6
NIL9311	2017	Cd	1.491 ± 0.160	1.981 ± 0.294	1.736 ± 0.200	35.92	< .*0001*	0.25	0.00	0.01	17.9
		HTD	106.7 ± 2.0	107.5 ± 1.4	n.a	2.99	*0.0212*	0.50			6.8
NIL611	2017	Cd	0.791 ± 0.081	0.527 ± 0.078	0.800 ± 0.095	101.48	< .*0001*	− 0.13	0.14	− 1.09	37.1
		HTD	89.0 ± 2.7	75.0 ± 2.1	n.a	274.29	< .*0001*	− 7.0			84.3
NIL819	2017	Cd	0.820 ± 0.092	0.392 ± 0.118	0.765 ± 0.113	137.42	< .*0001*	− 0.21	0.17	− 0.79	45.4
		HTD	92.0 ± 1.9	73.0 ± 1.6	n.a	1574.57	< .*0001*	− 9.5			96.6
IRAT129	2017	Cd	0.465 ± 0.107								
9311	2017	Cd		1.838 ± 0.216							
H611	2017	Cd		0.412 ± 0.044							
H819	2017	Cd		0.436 ± 0.062							

HTD is the days to heading. *Japonica* and *indica* are lines carrying homozygous *OsHMA3-OsNramp5-OsNramp1* haplotype from IRAT129 and *indica* variety (9311, H611 or H819), respectively. H, the lines carrying heterozygous *OsHMA3-OsNramp5-OsNramp1* haplotype. A, additive effect of replacing *OsHMA3-OsNramp5-OsNramp1*^*ind*^ haplotype with *OsHMA3-OsNramp5-OsNramp1*^*jap*^ haplotype. D, degree of dominance. *R*^2^, the proportion of phenotypic variance explained by *OsHMA3-OsNramp5-OsNramp1.*

### Validation of ***OsHMA3***-***OsNramp5***-***OsNramp1***^***jap***^ effects in the ***indica*** genetic background

Three sets of NILs of *OsHMA3*-*OsNramp5*-*OsNramp1* in an *indica* genetic background replaced with of 9311 (an elite two-line middle-season *indica* hybrid restorer in China), and H611 and H811 (two elite two-line early-season *indica* hybrid restorers in China) were developed to validate the practicality and effectiveness of *OsHMA3*-*OsNramp5*-*OsNramp1*^*jap*^ in molecular marker-assisted improvement of *indica* rice with low Cd-accumulating ability. Discrete distributions of Cd concentration were not observed in the three sets of NILs, but the frequency distributions were distinguishable among the different genotypes. *OsHMA3*-*OsNramp5*-*OsNramp1*^*jap*^ homozygous, *OsHMA3*-*OsNramp5*-*OsNramp1*^*ind*^ homozygous and heterozygous lines tended to have lower, higher and higher values in NIL9311, whereas it exhibited the opposite response in NIL611 and NIL819. These results indicated that different genotypic groups of NIL9311, NIL611 and NIL819 might carry different alleles for Cd, whereas *OsHMA3*-*OsNramp5*-*OsNramp1*^*jap*^ in NIL611 and NIL819 has a different genetic effect direction from that in NIL9311. Two-way ANOVAs were used to examine differences among the three genotypic groups in each of the two NIL sets. As expected, major allele effects were detected for Cd in all the three NIL sets and explained 17.9%, 37.1% and 45.4% of the phenotypic variance in NIL9311, NIL611 and NIL819, respectively. The *OsHMA3*-*OsNramp5*-*OsNramp1*^*jap*^ haplotype decreased Cd concentration in NIL9311 by 0.25 mg/kg (Table 3). However, in contrast to the results from RIL and NIL9311, the favourable Cd-decreasing allele was found in *indica* cultivars H611 and H819, and the *OsHMA3*-*OsNramp5*-*OsNramp1*^*jap*^ haplotype increased Cd by 0.13 mg/kg in NIL611 and 0.21 mg/kg in NIL819 (Table 3).

Major effects were detected for HTD in NIL611 and NIL819. The enhancing alleles were all from IRAT129; they increased HTD by 7.0 and 9.5 days and explained 84.3% and 96.6% of the phenotypic variance in NIL611 and NIL819, respectively. A minor effect was detected for HTD in NIL9311, and the enhancing allele came from 9311, increasing HTD by 0.5 days and explaining 6.8% of the phenotypic variance. These results indicated that the segment containing *OsHMA3*-*OsNramp5*-*OsNramp1* from IRAT129 has major effects on HTD in early-season *indica* rice and minor effects in middle-season *indica* rice and the directions of the genetic effects are opposite.

### Heading date and yield potential gene (*Ghd7*) analysis of NIL population

**Figure 2:**
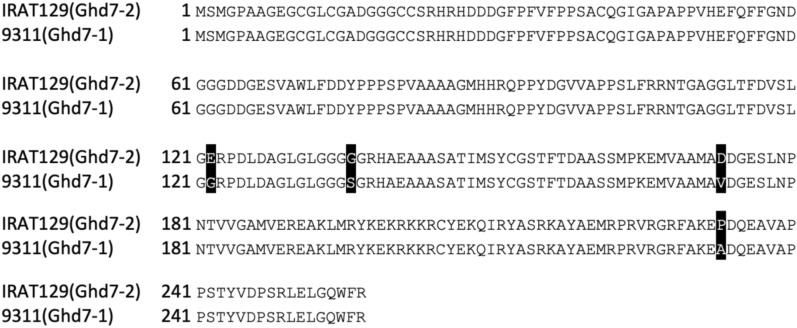
The predicted protein sequences of GHD7 for IRAT129, 9311, H611 and H819.

A cloned gene *Ghd7*, an important regulator of heading date and yield potential in rice, which plays a crucial role in increasing productivity and adaptability of rice globally is located close to *OsNramp1*, within a physical distance of about 181.5 kb (Chr.7: 8,970,856–9,152,377), according to the Rice Genome Annotation Project Database^23,24^. To investigate the role of *Ghd7* gene on the expression of *OsHMA3*-*OsNramp5*-*OsNramp1*^*jap*^ in early-season and middle-season *indica* genetic backgrounds, we obtained the *Ghd7* sequence of IRAT129, H611, H819 and 9311. Comparison of the predicted protein sequences identified three alleles, equivalent to *Ghd7-0*, *Ghd7-1* and *Ghd7-2* in a previous study^24^. IRAT129 and 9311 carried a functionally weaker allele of *Ghd7-2* and a fully functional allele of *Ghd7-1*, respectively (Fig. 2). The *Ghd7* locus of H611 and H819 was completely deleted (*Ghd7-0*). The genotyping of the three sets of NILs with an intragenic marker for *Ghd7* showed that the segment of *OsHMA3*-*OsNramp5*-*OsNramp1*^*jap*^ also contained *Ghd7*^*jap*^ in three sets of NILs. The HTD effects in early-season and middle-season *indica* rice could be a result from the effects of different *Ghd7* alleles.

### Genetic background and agronomic traits of improved 9311 with the ***OsHMA3***-***OsNramp5***-***OsNramp1***-***Ghd7***^***jap***^ haplotype

**Figure 3:**
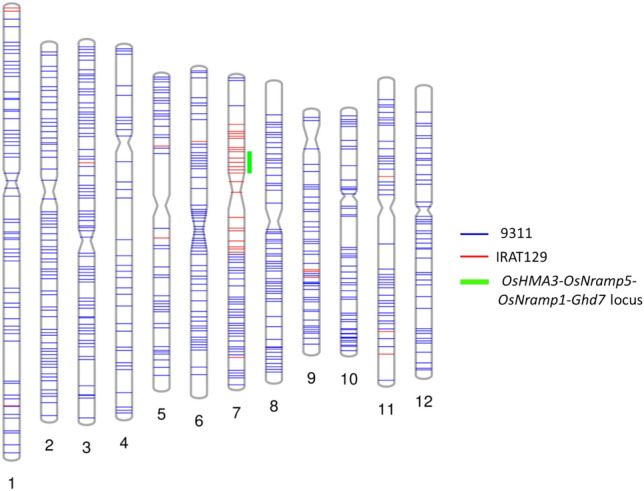
Graphical genotype maps of the improved 9311 (NIL9311-58). NIL9311-58 with the *OsHMA3-OsNramp5-OsNramp1-Ghd7*^*jap*^ haplotype was genotyped using 1.2 K SNP markers. Six hundred and thirty polymorphic SNPs between bi-parents were used for the construction of the genotype maps.

Eight improved homozygous plants from the NIL9311 population were genotyped using high-density SNP markers to check the genome recovery rate of the recurrent parent in the improved lines. Of the 1125 SNP markers covering 12 chromosomes, 630 SNPs showed polymorphism between the donor IRAT129 and recipient 9311. The selected improved plants showed a high genome recovery rate of the recurrent parent, at 90.4–91.6% (Table 4). A graphical genotype map of NIL9311-58 was constructed based on the SNP genotyping results (Fig. 3), assuming that the size of the target chromosome segment containing the *OsHMA3-OsNramp5-OsNramp1*^*jap*^ was approximately 14.3 Mb. The genetic background of NIL9311-58 was also analysed with 48 SSR makers, which are used for the identification of rice varieties (NY/T 1433-2014)^26^. The result showed only one polymorphic SSR marker RM542 (Chr. 7: 12,712,017–12,712,178), neighbouring *OsHMA3*-*OsNramp5*-*OsNramp1*-*Ghd7* (Chr. 7: 7,405,745–9,155,030)^27^, was identified between improved 9311 (NIL9311-58) and original 9311.

**Table 4:** Genome recovery rate of eight improved homozygous plants from NIL9311 population.

Line no	No. of SNP	No. of SNP allele			Genome ratio (%)	
		Recipient	Donor	Hetero	Recipient	Donor
NIL9311-11	617	558	56	3	90.7	9.3
NIL9311-12	619	560	56	3	90.7	9.3
NIL9311-21	620	561	56	3	90.7	9.3
NIL9311-54	619	563	53	3	91.2	8.8
NIL9311-58	617	565	52	0	91.6	8.4
NIL9311-62	619	560	57	2	90.6	9.4
NIL9311-61	619	562	54	3	91.0	9.0
NIL9311-62	618	556	57	5	90.4	9.6

To test whether the *OsHMA3*-*OsNramp5*-*OsNramp1*-*Ghd7*^*jap*^ haplotype has a negative effect on the yield, the agronomic traits and grain metallic element of eight lines derived from NIL9311-58 (improved 9311carrying *OsHMA3-OsNramp5-OsNramp1*^*jap*^ with favourable agronomic performance and lower Cd accumulation in the grain) and the control 9311 were evaluated in the middle rice-cropping season at Niangxiang in 2018. The improved 9311 showed a significant increase in Mn concentration, panicle numbers and yield, and a significant decrease in DTH and grain Cd accumulation compared with the control 9311 (Table 5). Overall, the improved 9311 had no negative effect on yield, whereas it decreased grain Cd accumulation by about 31.8% and increased Mn accumulation by about 21.2%.

**Table 5:** Agronomic performance of improved 9311 (NIL9311-58) lines carrying *OsHMA3-OsNramp5-OsNramp1*^*jap*^ in the genetic background of 9311.

Genotype	NL	DTH	PH	PN	NGP	SF (%)	TGW (g)	YP (g)	Cd (mg/kg)	Fe (mg/kg)	Mn (mg/kg)	Cu (mg/kg)	Zn (mg/kg)
9311	5	92.0 ± 0.3	119.0 ± 1.1	8.3 ± 1.1	208.7 ± 7.1	89.1 ± 7.8	29.9 ± 1.0	36.7 ± 2.7	1.57 ± 0.25	19.97 ± 0.01	42.73 ± 0.75	3.29 ± 0.26	31.7 ± 0.70
NIL9311-58	8	89.4 ± 1.5*	117.9 ± 2.6	9.6 ± 1.2*	213.9 ± 15.2	88.1 ± 1.9	29.1 ± 0.6	38.4 ± 2.1*	1.07 ± 0.12*	20.8 ± 0.07	51.77 ± 2.17*	3.45 ± 0.20	31.37 ± 1.31

NL: number of lines; DTH: days to heading; PH: plant height; PN: number of panicles per plant; NGP: number of grains per panicle; SF: spikelet fertility; TGW: 1000-grain weight; YP: yield per plant; Cd, Fe, Mn, Cu, Zn: Cd, Fe, Mn, Cu and Zn content in brown grains.

*Significance at *P* < 0.01.

### Haplotype and expression of *OsHMA3*, *OsNramp1* and *OsNramp5* in IRAT129, 9311, NIL9311-58, H611 and H819

**Figure 4:**
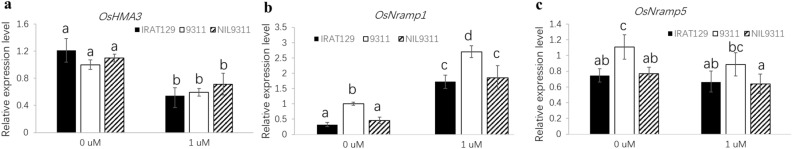
Relative expression of *OsHMA3* (**a**), *OsNramp1* (**b**) and *OsNramp5* (**c**) in rice roots of different lines in the presence or absence of 1 μM Cd. Plants of IRAT129, 9311 and NIL9311-58 were treated with and without 1 μM Cd for 24 h. Data were means ± SD (n = 3). Means with different letters are significantly different at *P* < 0.05 (one-way ANOVA, Tukey HSD test).

At least eight alleles (I to VIII) of *OsHMA3* have identified in previous reports^25^. The coding regions of *OsHMA3* of IRTA129, 9311, H611 and H819 were therefore sequenced. Results indicated that 9311, H611 and H819 have the same functional allele of type V, whereas IRTA129 did not belong to any of the eight haplotypes, which has a single amino acid mutation at 550th position with IIe being substituted by Val compared to type V allele (designated as type X here) (Table 6). Liu et al. reported that the expression difference of *OsHMA3*, which due to natural variation in the promoter of *OsHMA3*, contributes to differential grain cadmium accumulation between *indica* and *japonica* rice^20^. We therefore compared the expression level of *OsHMA3* in roots of IRTA129, 9311 and NIL9311-58. In inconsistent with the results of Liu et al., there were no significant differences in *OsHMA3* expression between the three cultivars tested in either the absence or the presence of Cd. The presence of Cd tended to decrease the expression of *OsHMA3*, and the effect was significant in all tested lines (Fig. 4a). Sequence variations in the about 2 kb *OsHMA3* promoter were therefore investigated. The results showed that promoter sequences in 9311, H611 and H819 are the same as that of type 1 (*indica* type) in the research of Liu et al^20^. Thirteen nucleotide variations were identified between type 1 and IRTA129 promoter (Table S1). Liu et al. revealed that the differential transcriptional activity of *OsHMA3* promoter between *indica* and *japonica* could be attributed to the seven nucleotide changes occurring in the region between − 683 and − 557 bp^20^. However, there was no nucleotide variation between type1^*indica*^ and IRTA129 ^*japonica*^ promoter.

**Table 6:** OsHMA3 haplotypes of 9311, H611 and IRAT129.

Cultivar	Subspecies	Amino acid position												HMA3 haplotype*	Functional*
		80	299	380	550	614	638	678	728	736	752	775	826–878		
Nipponbare^a^	*Japonica*	R	F	S	V	S	A	C	T	G	V	E		I	Yes
Akita 64^b^	*Japonica*	R	F	S	V	S	A	C	T	G	V	E		I	Yes
Dular^a^	*Aus*	R	F	S	V	S	A	C	T	C	V	E		IV	Yes
Fengaizhan^a^	*Indica*	R	L	S	I	G	A	R	T	G	A	E		V	Yes
Cho-Ko-Koku^b^	*Indica*	H	L	S	V	D	V	R	T	G	A	E	Deletion	VIII	No
9311	*Indica*	R	L	S	I	G	A	R	T	G	A	E		V	Yes
H611	*Indica*	R	L	S	I	G	A	R	T	G	A	E		V	Yes
H819	*Indica*	R	L	S	I	G	A	R	T	G	A	E		V	Yes
IRAT129	*Japonica*	R	L	S	V	G	A	R	T	G	A	E		X	

^a^The data was cited from Yan et al^24^.

^b^The data was cited from Miyadate et al.^13^.

The coding regions of *OsNramp1* and *OsNramp5* of IRTA129, 9311, H611 and H819 were also sequenced. Results indicated that 9311, H611 and H819 have the different allele of *OsNramp1* with that of IRTA129, which has three single amino acid mutation at 34th, 252th and 512th position, respectively (Table S2). Consistent with results of Takahashi et al.^14^ and Chang et al.^21^, the level of *OsNramp1* expression in the roots of *indica* 9311 was higher than that of *japonica* IRAT129 and NIL9311-58 in either the absence or the presence of Cd. The presence of Cd tended to increase the expression of *Nramp1*, and the effect was significant in all tested lines (Fig. 4b). Sequence variations in the about 2 kb *OsNramp1* promoter were therefore investigated. The results showed that promoter sequences in 9311, H611 and H819 are the same as that of type 1 (*indica* type) in the research of Liu et al.^20^. Nineteen nucleotide variations including the 406 bp InDel variation were identified between IRTA129 and 9311 promoters (Table S3). The 406 bp were missing in 9311, as well as in H611 and H819. Sequencing analysis results showed that 9311, H611, H819 and IRTA129 have the same allele of *OsNramp5* with no amino acid variation. Similar results with the research of Liu et al.^22^, the level of *OsNramp5* expression in the roots of 9311 was significantly higher than that of IRAT129 and NIL9311-58 in either the absence or the presence of Cd (Fig. 4c). Twelve nucleotide variations were found in the promoter region (Table S4). These sequence variations in the promoter region could lead to the expression level difference of *OsNramp1* and *OsNramp5* between 9311 and NIL9311-58.

## Discussion

Accumulation of Cd in crops is a serious agricultural issue. Reducing Cd concentration in the edible parts of crops through breeding is a promising option for decreasing risks to human health without any additional cost to farmers. The incorporation of favourable alleles of major Cd accumulation QTLs/genes could be an important measure to decrease the grain Cd concentration of rice. *Japonica* rice generally accumulates lower amounts of Cd in the grain than do *indica* and *aus* cultivars. Three genes (*OsHMA3*, *OsNramp5* and *OsNramp1*), located in a region of about 1565 kb on chromosome 7 (physical location: 7,405,745–8,970,856), have previously been identified as playing critical roles in Cd accumulation in rice. However, there is no evidence that *OsHMA3*, *OsNramp5* or *OsNramp1* is/are the casual gene(s) for general differences in Cd accumulation between *indica* and *japonica* rice, and there is no previous report on the use of *OsHMA3*, *OsNramp5* or *OsNramp1* from low Cd accumulation *japonica* cultivars in *indica* improvement for low Cd accumulation. In this study, we investigated the role of genomic segment covering *OsHMA3*-*OsNramp5*-*OsNramp1*, by introgressing it in an *indica* breeding programme aimed at decreasing potential grain Cd pollution risk in elite *indica* cultivars. For the evaluation of the effect on Cd accumulation, the *OsHMA3*-*OsNramp5*-*OsNramp1*^*jap*^ haplotype was tested in an *indica–japonica* cross background and a different season-type *indica* cultivar background using RILs and NILs population over two cropping seasons.

In this study, *OsHMA3*-*OsNramp5*-*OsNramp1* was found to explain 8.6–10.9% of the grain Cd concentration variance in RILs derived from *indica*/*japonica* crossing (9311 × IRAT129) in two cropping seasons, and the favourable allele came from *japonica*, as expected. For the further evaluation of the *OsHMA3*-*OsNramp5*-*OsNramp1*^*jap*^ haplotype in the *indica* breeding programme, we removed the *japonica* donor genome from the breeding lines. NILs of *OsHMA3*-*OsNramp5*-*OsNramp1* in different *indica* backgrounds (9311, H611 and H819) were developed by advanced-backcross and MAS. 9311 is an elite male parent of two-line middle-season hybrid rice, as well as a favourable *indica* variety in China. 9311 is the male parent of Liangyoupei9 and Y-liangyou1, which are representative varieties of the first- and second-stage super-hybrids in China. Liangyoupei9 was the hybrid rice variety with the largest yearly promotion area from 2002 to 2007, except in 2004, and Y-liangyou-1 had the largest yearly promotion area from 2010 to 2013 in China. However, 9311 and the hybrid rice varieties derived from 9311 have high Cd accumulation, especially in grains. H611 and H819 are the elite male parents of two-line early-season hybrid rice. Zhulinagyou819, the combination of Zhu1S and H819, is the first hybrid rice to be identified as a low Cd accumulation variety in Hanan province, China. The *OsHMA3*-*OsNramp5*-*OsNramp1*^*jap*^ haplotype significantly decreased grain Cd concentration in a middle-season *indica* 9311 genetic background. The homozygotes of *OsHMA3*-*OsNramp5*-*OsNramp1*^*jap*^ accumulated 24.7% less Cd in grains than did homozygotes of *OsHMA3*-*OsNramp5*-*OsNramp1*^*ind*^. These results indicate that the *OsHMA3*-*OsNramp5*-*OsNramp1*^*jap*^ haplotype can be used in middle-season *indica* improvement for low Cd concentration in the grains. Among the 23 homozygous lines of *OsHMA3*-*OsNramp5*-*OsNramp1*^*jap*^, NIL 9311-58 showed the lowest Cd concentration in the grain, 40.7% less than 9311. Eight lines (improved 9311) were derived from NIL 9311-58 to evaluate whether the *OsHMA3*-*OsNramp5*-*OsNramp1*-*Ghd7*^*jap*^ haplotype had negative effects on yield performance. The improved 9311 had significantly decreased grain Cd accumulation, by about 31.8%, and significantly increased yield, of about 4.6%. This improved 9311 line, a near isogenic line, is similar to 9311 variety in all agronomic aspects except low Cd accumulation in the grain. Therefore, the improved 9311 could replace 9311 for rice production in areas where Cd pollution is a potential threat. However, in contrast to the performance in middle-season *indica* background, the *OsHMA3*-*OsNramp5*-*OsNramp1*^*jap*^ haplotype significantly increased grain Cd concentration in two early-season *indica* (H611 and H819) background. In addition, major effects were detected for HTD in NIL611 and NIL819, and the *OsHMA3*-*OsNramp5*-*OsNramp1*^*jap*^ haplotype has significantly increased HTD. This observation might imply the existence of a linkage-drag or pleiotropic effect of the segment containing *OsHMA3*-*OsNramp5*-*OsNramp1* for grain Cd concentration in the early-season *indica* background. Some previous works also reported the QTLs related to grain Cd concentration overlap or contained the QTLs/genes for DTH^28,29^. The pleiotropic effect of QTLs for grain Cd concentration and/or water management difference of the genotypes with different heading time in one trial plot is/are probably the main reason(s), which caused the experiment results above^30,31^.

Beside *OsHMA3*-*OsNramp5*-*OsNramp1*, an HTD gene, *Ghd7*, is located at a physical distance of about 181.5 kb from *OsNramp1*. Xue et al. (2008) reported that *Ghd7-1* is a fully functional allele and *Ghd7-2* is a weak functional allele. The *Ghd7* locus was completely deleted in early-season rice (*Ghd7-0*). Our study showed that *Ghd7* of *japonica* IRAT129, middle-season *indica* 9311, and early-season *indica* H611 and H819 was equivalent to the *Ghd7-2*, *Ghd7-1* and *Ghd7-0* type, respectively. All three NIL populations carried *OsHMA3*-*OsNramp5*-*OsNramp1-Ghd7*^*ind/jap*^. A minor HTD effect was identified in the NIL9311 population, possibly because of the effects of the *Ghd7-1* and *Ghd7-2* alleles on HTD. Linkage-drag with *Ghd7* could be the reason for the *OsHMA3*-*OsNramp5*-*OsNramp1*^*jap*^ haplotype to significantly increase HTD in early-season *indica* background. The linkage-drag with *Ghd7* should be broken if the *OsHMA3*-*OsNramp5*-*OsNramp1*^*jap*^ haplotype is to be used in early-season *indica* improvement. The improved early-season *indica* lines with *OsHMA3*-*OsNramp5*-*OsNramp1-Ghd7*^*jap*^ showed late heading, and these cannot be cropped as early-season *indica*. *Ghd7* is located in the centromeric region of chromosome 7, therefore the recombination rate is considerably low. Xue et al. (2008) reported that the recombination rate around *Ghd7* region is about 37-fold lower than the genome average of approximately 200 kb/cM. A large segregating population (≥ 4077 plants, theoretically) should be developed to identify recombination events between *OsNramp1* and *Ghd7*, if we are to use the *OsHMA3*-*OsNramp5*-*OsNramp1*^*jap*^ haplotype to improve early-season *indica* for low Cd grain concentration. The target chromosome segment of *OsHMA3*-*OsNramp5*-*OsNramp1* was selected using three intragenic markers for *OsHMA3*, *OsNramp5* and *OsNramp1* and none recombinant was used. To further investigate the effect of low Cd accumulation in grain is from one or several gene(s) of *OsHMA3*, *OsNramp5* and *OsNramp1*, the recombinants should be identified from the large segregating population derived from heterozygotes of *OsHMA3*-*OsNramp5*-*OsNramp1*.

A novel allele of *OsHMA3* was identified from a wide-compatibility *japonica* cultivar IRAT129 with low Cd-accumulating ability. Nucleotide variations were identified between IRAT129 and higher Cd-accumulating *indica* variety (9311, H611 and H819) promoter, but there were no significant differences in *OsHMA3* expression between 9311 and NIL9311 tested in either the absence or the presence of Cd. Whether *OsHMA3* allele from IRAT129 has stronger function on decreasing Cd-accumulating ability need be further studied. The expressions of *OsNramp1* and *OsNramp5* in roots all were lower in lower Cd-accumulating variety/line (IRAT129 and NIL9311-58) than that in higher Cd-accumulating variety (9311). The 400 bp deletion in the promoter region of *OsNRAMP1* and sequence variations identified in the promoter region of *OsNRAMP5* could lead to differences in the expression level of *OsNramp1* and *OsNramp5*, as well as in Cd accumulation in the grain between 9311 and NIL9311-58. Our results provide evidence that segment covering *OsHMA3-OsNramp5-OsNramp1* on chromosome 7 of a *japonica* cultivar IRAT129 can be used in *indica* varieties improvement with accumulating low levels of Cd. But, the linkage-drag between *OsHMA3-OsNramp5-OsNramp1* and *Ghd7* should be broken down by large segregating populations or gene editing in early-season *indica* improvement. The improved 9311 with introgression of *OsHMA3-OsNramp5-OsNramp1*^*IRAT129*^ showed significant decrease in grain Cd accumulation with no yield loss. However, the improved 9311 still cannot produce Cd-free grain (< 0.2 or 0.4 mg/kg) in mild Cd-polluted paddy field with intermittent irrigation management. Assembling large numbers of favourable low-Cd gene/QTL alleles or edit Cd accumulating genes (such as *OsNramp5*, etc.) could be further studied for improving rice with Cd-free grain.

## Methods

### Plant materials

A RIL (F_7_) population consisting of 133 lines was generated from an F_1_ hybrid between 9311 (an elite two-line middle-season *indica* hybrid restorer with high Cd-accumulating ability in China) and IRAT129 (a wide-compatibility *japonica* cultivar with low Cd-accumulating ability) by single seed descent.

Three sets of NILs including the genomic region covering the *OsHMA3*, *OsNramp5* and *OsNramp1* genes, with different genetic backgrounds, were also developed. IRAT129 was the paternal parent, and the IRTA129-type *OsHMA3*-*OsNramp5*- *OsNramp1* haplotype (*OsHMA3*-*OsNramp5*- *OsNramp1*^*jap*^) donor was crossed with two elite two-line early-season *indica* hybrid restorers (H611 and H811) and an elite two-line middle-season *indica* hybrid restorer (9311) as maternal recipients. The resulting F_1_ plants were backcrossed with the recipients. Plants heterozygous for the target region in the progeny were selected using molecular markers and crossed to recipients. Finally, BC_3_F_1_ plants heterozygous for the target region were selected to produce BC_3_F_2_, which were genotyped using molecular markers, and *japonica*-type homozygotes (*OsHMA3*-*OsNramp5*-*OsNramp1*^*jap*^), *indica*-type homozygotes (*OsHMA3*-*OsNramp5*-*OsNramp1*^*ind*^) homozygotes and heterozygotes (*OsHMA3*-*OsNramp5*-*OsNramp1*^*jap*^/*OsHMA3*-*OsNramp5*-*OsNramp1*^*ind*^) were selected. The three sets of NILs were derived from the selected seeds and named NIL611, NIL819 and NIL9311 with H611, H819 and 9311 genetic backgrounds, respectively. Eight plants selected from the NIL9311-58 line were named improved 9311.

### DNA preparation, molecular marker development, sequencing and expression analysis

Genomic DNA was extracted from 2-week-old seedlings using the CTAB method as described by Murray and Thomson^32^. Three newly developed intragenic markers were developed (Table 2) based on publicly available rice genome sequences^23,33^. Gene cloning and sequencing were done following the methods described in Yan et al.^25^ and Lu et al.^34^. Quantitative reverse transcription PCR (qRT-PCR) analysis was done following the methods described in Yan et al^25^. Primers for cloning, sequencing and qRT-PCR in this study were listed in Table S5.

### Background genotyping and construction of a graphical genotype map

Genomic DNA was prepared using the CTAB method as described by Murray and Thomson^31^. A 1.2 K multiplex-PCR panel, which was based on GBTS platform from MolBreeding Biotechnol (http://www.molbreeding.com), was employed for background genotyping. A total of 48 SSR makers were also used in background genotyping, following the protocol of the identification of rice varieties by SSR marker method (NY/T 1433–2014).

### Field experiments and phenotypic analysis

Field experiments were conducted in a Cd-polluted paddy field (Niangxiang, China) in the 2016, 2017 and 2018 rice-growing seasons. The soil Cd concentration was around 0.40 mg/kg with *pH* 6.4. The irrigation water Cd concentration was about 0.027 ug/L. Field trials of RILs were conducted in the 2016 and 2017 middle-season rice growing seasons. NILs of NIL611 and NIL819 were conducted in the 2017 early-season rice growing season, as well as NIL9311 in the 2017 middle-season rice growing season. The experiments were arranged in a randomised complete block design with three replicates, with 10 plants per line. Field management followed normal agricultural practices, except that intermittent irrigation was adopted to maximise the phenotypic differences. DTH was counted from seed sowing to the flowering of 50% of the plants of each line. At maturity, the middle eight plants of each line in each replication were harvested, and the Cd concentrations of brown rice were determined using NX-100FA.

The 9311 and improved 9311 lines were cropped in the 2018 middle-season rice growing season, arranged in a randomised complete block design with three replicates and 40 hills per replicate. Field management followed that of the 2016 and 2017 field experiments. Days to heading (DTH) was counted from seed sowing to the flowering of 50% of the plants of each line. The plant height (PH) of five normal plants in the middle of each plot was measured at the fully mature stage, and these five plants were harvested to evaluate various agronomic traits in the laboratory, including the number of grains per panicle (NGP), spikelet fertility (SF), 1000-grain weight (TGW) and yield per plant (YP).

### Statistical analysis

The SAS (V8.01) procedure GLM was used to evaluate phenotypic variation among the different genotypic groups in the RILs and NILs^35^.

## Supplementary Information

Supplementary Information

## Data Availability

The datasets supporting the conclusions of this article are included within the article and its additional files. The seeds of RILs, NILs and the parents are available from the corresponding author on reasonable request.
