# Supplementary material for: Validating a segment on chromosome 7 of japonica for establishing low-cadmium accumulating indica rice variety
Source: Sci Rep. 2021 Mar 15;11:6053. doi: 10.1038/s41598-021-85324-0 (PMC7961068; doi:10.1038/s41598-021-85324-0)
Supplement: Supplementary file 1 — Supplementary Information [file 41598_2021_85324_MOESM1_ESM.docx]

Supplementary Information File

Validating a segment on Chromosome 7 of *japonica* for establishing low-cadmium accumulating *indica* rice variety

Kai Wang^1,2,3,+^, Tian-ze Yan^1,+^, Shi-long Xu^1,+^, Xu Yan^1,4^, Qun-feng Zhou^1^, Xin-hui Zhao^1^, Yan-feng Li^1^, Zhong-xiu Wu^1,2^, Peng Qin^1^, Chen-jian Fu^1^, Jun Fu^1^, Yan-biao Zhou^1^ & Yuan-zhu Yang^1,2,3*^

^1^Key Laboratory of Southern Rice Innovation & Improvement, Ministry of Agriculture and Rural Affairs, Hunan Engineering Laboratory of Disease and Pest Resistant Rice Breeding, Yuan Longping High-Tech Agriculture Co., Ltd., Changsha 410128, China.

^2^ State Key Laboratory of Hybrid Rice, Hunan Hybrid Rice Research Center, Changsha, 410125, China.

^3^Department of Botany, College of Life Sciences, Hunan Normal University, Changsha 410081, China.

^4^ College of Plant Science & Technology, Huazhong Agricultural University, Wuhan 430070, China

^*^Corresponding author, E-mail: [yzhuyah@163.com](mailto:yzhuyah@163.com)

^+^These authors contributed equally to this work.

Running title: Establishing low-cadmium accumulating *indica* variety improvement

**Table S1.** Sequence variations in *OsHMA3* promoter of different rice cultivars.

| **Variations Position** | **IRAT129** | **93-11** | **H611** | **H819** | **Nipponbare** |
| --- | --- | --- | --- | --- | --- |
| -200 | C | T | T | T | T |
| -321 | A | T | T | T | T |
| -557 | A | A | A | A | T |
| -558 | A | A | A | A | T |
| -576 | G | G | G | G | A |
| -597 | G | G | G | G | T |
| -611 | G | G | G | G | A |
| -620 | G | G | G | G | A |
| -683 | G | G | G | G | T |
| -820 | G | A | A | A | G |
| -991 | A | G | G | G | A |
| -1211 | G | A | A | A | A |
| -1419 | C | G | G | G | G |
| -1676 | - | A | A | A | - |
| -1677 | - | C | C | C | - |
| -1712 | G | T | T | T | G |
| -1745 | A | A | A | A | C |
| -1758 | G | A | A | A | G |
| -1778 | T | - | - | - | A |
| -1779 | T | A | A | A | A |
| -1805 | T | T | T | T | C |
| -1855 | A | G | G | G | G |
| -1981 | C | C | C | C | A |

**Table S2.** OsNramp1 haplotypes of 9311, H611, H819 and IRAT129.

| **Cultivar** | **Subspecies** | **Amino Acid Position** | | |
| --- | --- | --- | --- | --- |
|  |  | **34** | **252** | **512** |
| Nipponbare | *Japonica* | R | V | H |
| IRAT129 | *Japonica* | R | V | H |
| 9311 | *Indica* | K | A | D |
| H611 | *Indica* | K | A | D |
| H819 | *Indica* | K | A | D |

**Table S3.** Sequence variations in *OsNramp1* promoter of different rice cultivars.

| **Variations Position** | **IRAT129** | **93-11** | **H611** | **H819** | **Nipponbare** |
| --- | --- | --- | --- | --- | --- |
| -297 | G | C | C | C | G |
| -434 | T | T | C | C | T |
| -456 | A | G | G | G | A |
| -488 | G | A | G | G | G |
| -528 | C | G | C | C | C |
| -721 | T | G | G | G | T |
| -733 | G | A | G | G | G |
| -777 | C | T | T | T | C |
| -847 | T | C | T | T | T |
| -1002 | A | G | G | G | A |
| -1033 | A | C | C | C | A |
| -1046 | C | T | T | T | C |
| -1060 | T | - | T | T | T |
| -1077 | A | G | A | A | A |
| -1100 | G | T | T | T | G |
| -1104 | T | 406bp deletion | 406bp deletion | 406bp deletion | T |
| -1154 | T |  |  |  | C |
| -1509 | C |  |  |  | C |
| -1703 | A | T | A | A | A |
| -1862 | A | G | G | G | A |
| -1883 | G | T | T | T | G |
| -1896 | G | A | G | G | G |
| -1912 | C | C | T | T | C |
| -1932 | G | G | G | G | - |

**Table S4.** Sequence variations in *OsNramp5* promoter of different rice cultivars.

| **Variations Position** | **IRAT129** | **93-11** | **H611** | **H819** | **Nipponbare** |
| --- | --- | --- | --- | --- | --- |
| -50 | C | - | - | - | C |
| -832 | T | C | C | C | T |
| -930 | G | T | T | T | G |
| -1214 | T | T | T | T | C |
| -1365 | A | G | G | G | A |
| -1385 | C | T | T | T | C |
| -1586 | T | C | C | C | T |
| -1692 | C | T | T | T | C |
| -1725 | A | C | C | C | A |
| -1800 | T | A | A | A | T |
| -1866 | G | A | A | A | G |
| -1950 | T | C | C | C | T |

**Table S5.** Primers for cloning, sequencing and qRT-PCR in this study.

| Primer name | Forward primer (5'-3') | Reverse primer (5'-3') |
| --- | --- | --- |
| *OsHMA3*genome DNA | ACCATTGCCGGATTAGCTGT | CACGCTTGTGGCTATTGAGTG |
| *OsHMA3 promoter* | CGAGAATCGCGTGGAGGATTA | AATAGAGCCGTGGACATGCG |
| *OsNramp5*genome DNA (1) | AGCTCTAGCTTAGCCTGAAGAA | CCAGCACGTTCTGAACCACA |
| *OsNramp5*genome DNA (2) | TCGACACCTCCTCCTTCCTT | GAGCCACCTCCCCTCAAATG |
| *OsNramp5 promoter* | GCGCATGTATCATTTGTTGT | CTCACTGCTCTCTCTCTCAA |
| *OsNramp1*genome DNA (1) | GAGGATCTCCGCTCGAACTC | AACCGGAGGCTTGACTATGC |
| *OsNramp1*genome DNA (2) | CTGGCTCTAGCACGCTTCTT | TTTCAGCCGGTTATCACTGC |
| *OsNramp1 promoter* | GCCTACCCGCAGGATAATGG | GACATCGATGCGATCCCTCA |
| *OsHMA3*(qPCR) | TCCATCCAACCAAACCCGAAA | TGCCAATGTCCTTCTGTTCCCA |
| *OsNrmap5*(qPCR) | CAGCAGCAGTAAGAGCAAGATG | GTGCTCAGGAAGTACATGTTGAT |
| *OsNramp1*(qPCR) | CATCGGCATCGTGCTGTTC | TGGCTACCTGTGCTTTCTCG |
| *α-tubulin*(qPCR) | TCTTCCACCCTGAGCAGCTC | AACCTTGGAGACCAGTGCAG |
| *OsActin*(qPCR) | GACTCTGGTGATGGTGTCAGC | GGCTGGAAGAGGACCTCAGG |
